# Endovascular intervention with intravascular ultrasound guidance of very early dissection complication in transplant renal artery: a case report and literature review

**DOI:** 10.3389/fcvm.2024.1396998

**Published:** 2024-05-22

**Authors:** Vu Hoang Vu, Nghia Thuong Nguyen, Chinh Duc Nguyen, Khang Duong Nguyen, Binh Quang Truong

**Affiliations:** ^1^ Medicine Faculty, University of Medicine and Pharmacy at Ho Chi Minh City, Ho Chi Minh City, Vietnam; ^2^ Interventional Cardiology Department, University Medical Center Ho Chi Minh City, Ho Chi Minh City, Vietnam; ^3^ Interventional Cardiology Department, Cho Ray Hospital, Ho Chi Minh City, Vietnam; ^4^ Cardiovascular Center, Can Tho Stroke International Services, Can Tho, Vietnam

**Keywords:** intravascular ultrasound, kidney transplantation, transplant renal artery dissection, endovascular intervention, complication

## Abstract

**Background:**

Transplant renal artery dissection (TRAD) is a rare and serious event that can cause allograft dysfunction and eventually graft loss. Most cases are managed by operative repair. We report a case of TRAD in the early postoperative period, which was successfully managed with intravascular ultrasound-assisted endovascular intervention.

**Case presentation:**

A 38-year-old man underwent HLA-compatible living kidney transplantation. The allograft had one renal artery and vein, which were anastomosed to the internal iliac artery and external iliac vein, respectively. Doppler ultrasonography performed a day after the operation showed an increase in systolic blood velocity, with no observed urine output and raising a suspicion of arterial anastomotic stenosis. Angiography showed a donor renal artery dissection distal to the moderately stenosed anastomosis site with calcified atherosclerotic plaque confirmed by IVUS. The transplant renal artery lesion was intervened with a stent. After the intervention, Doppler US revealed that the blood flow of the renal artery was adequate without an increase in the systolic blood velocity. Urine output gradually returned after 3 weeks, and serum creatinine level was normalized after 2 months.

**Conclusions:**

Transplant recipients commonly have atherosclerosis and hypertension, which are risk factors for arterial dissection. Our case showed that endovascular intervention can replace surgery to repair very early vascular complications such as dissection and help patients avoid high-risk operations. Early diagnosis and IVUS-assisted intervention with experienced interventionists can save allograft dysfunction.

## 1 Introduction

Kidney transplantation is considered the best optimal treatment for end-stage renal disease (ESRD), associated with a lower mortality risk compared with dialysis and an improved quality of life. In the last decade, the number of kidney transplants has gradually increased worldwide (1, 2). Although surgical techniques have become more refined, there are still cases where complications occur during the perioperative period. The incidence of vascular problems is approximately 2%–3%, and the outcomes can be catastrophic, leading to allograft malfunction and ultimately the death of the patient with a transplanted kidney. Transplant renal artery dissection (TRAD) is an infrequent and severe event that poses a threat to the transplanted kidney (3, 4).

In the present case, a 38-year-old man receiving a living donor kidney with the transplanted renal artery anastomosed end-to-end to the internal iliac artery, TRAD occurred unexpectedly early in the transplanted renal artery. The patient received a timely diagnosis and intervention. Furthermore, the importance of IVUS was reaffirmed to diagnose and perform appropriate interventions for the salvage of allograft function. We also performed a literature review on this situation. We searched on PubMed with the following keywords: “dissection” and “renal artery implantation.” There were 331 results, of which 29 studies were included in the final review. The exclusion criteria included duplication, no reports of specific dissection, dissection before the implantation, dissection due to other causes, and no available full text.

## 2 Case presentation

**Figure 1:**
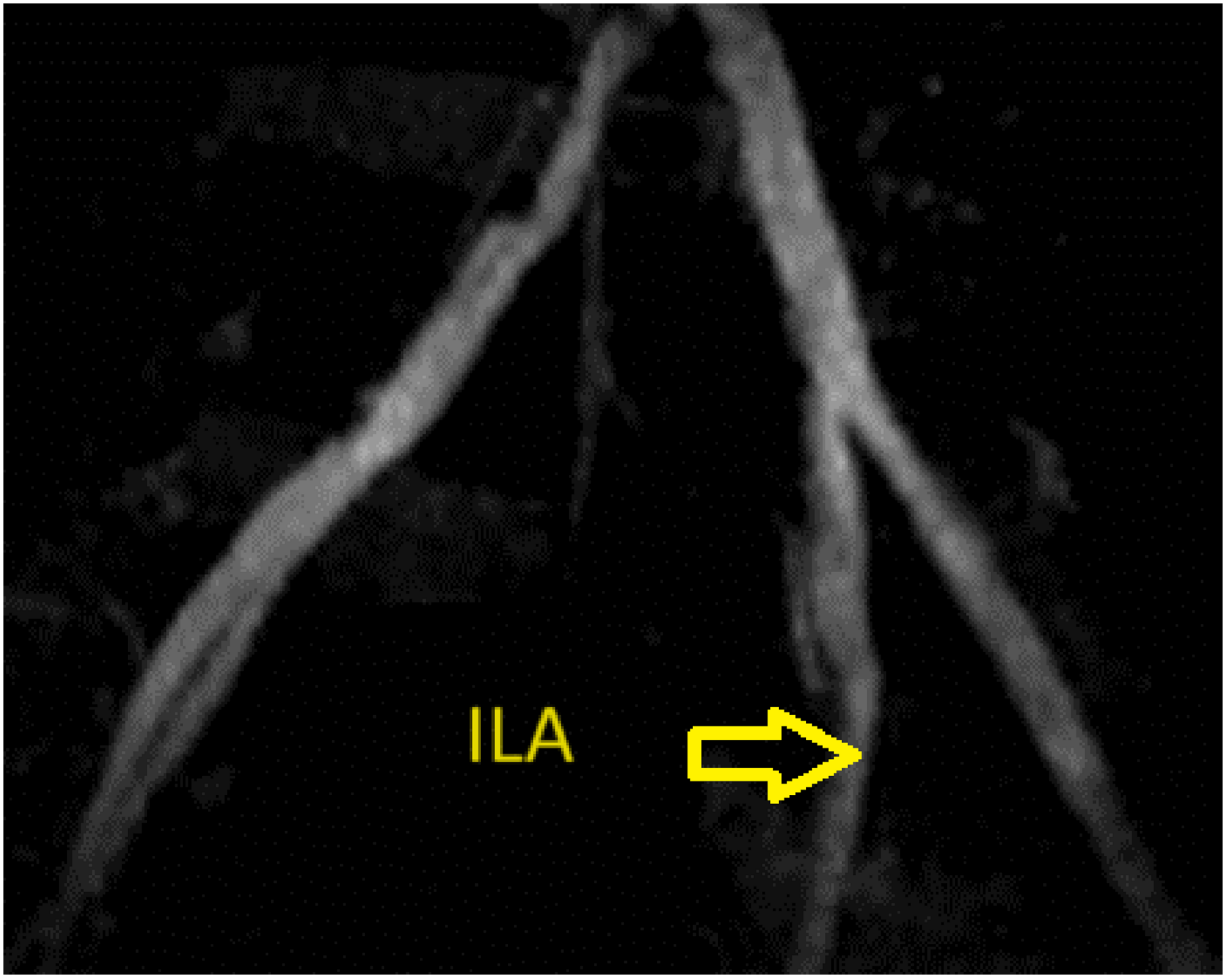
Representative images of the allograft obtained by preoperative enhanced computed tomography. There was mild stenosis of the left internal iliac artery.

A 38-year-old man was admitted to our hospital with clinical acute pulmonary edema and overload volume. His laboratory results showed proteinuria and a remarkable increase in serum creatinine level (11.4 mg/dl). After throughout investigations, he was finally diagnosed with idiopathic ESRD. The patient had undergone intermittent hemodialysis three times a week for 4 years and was hospitalized for scheduled living kidney transplantation. Preoperative evaluation of the iliac vessels of the recipient with MRI and Doppler ultrasound showed moderate stenosis of the right common iliac artery, severe stenosis of the right internal iliac artery, and moderate stenosis of the right femoral and popliteal floors. On the left side, there were mild stenosis of the left internal iliac artery and severe stenosis of the left femoral-popliteal floors (Figure 1). However, the patient experienced no symptoms.

He underwent a kidney transplant using a donated kidney from his 63-year-old mother. The right kidney was extracted, and the transplanted organ contained a solitary artery that exhibited no signs of atherosclerosis or stenosis. In this case, we chose to take the right kidney from the donor because the left kidney function was better on renal scintigraphy.

Due to substantial stenosis in the left femoral-popliteal artery, we decided against doing the surgery on the upstream external iliac artery. Therefore, the decision was made to select the left internal iliac artery instead. The transplanted artery and vein were anastomosed to the internal iliac artery and external iliac vein, respectively. The surgeon noted that there were atherosclerotic and calcified lesions in the left internal iliac artery at the anastomosis site, which made the surgical suture difficult. After releasing the artery clamp, the grafted renal artery pulsed weakly with lower amplitude compared to the internal iliac artery. Three bleeding points were detected at the site of venous anastomosis after releasing of vein clamp so the artery was clamped for another 30 min. Cold and warm ischemia time of kidney transplantation was 92 min and 86 min, respectively. Blood loss volume was 900 ml.

**Figure 2:**
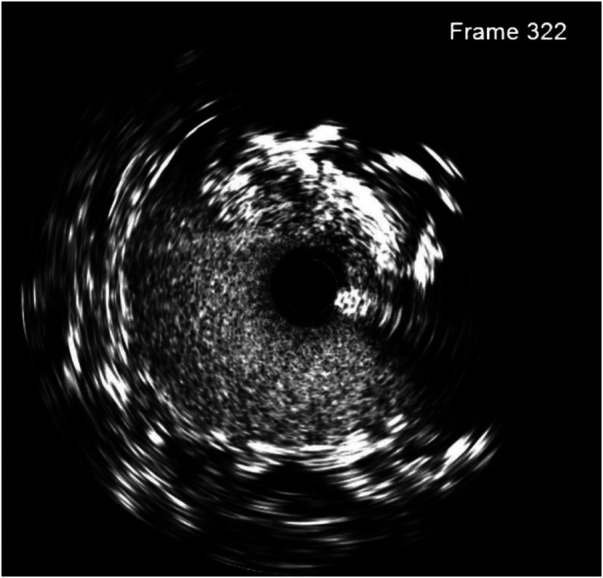
Intimal dissection (at 10 o'clock) was observed in the pre-transplantation IVUS image of the transplant artery.

**Figure 3:**
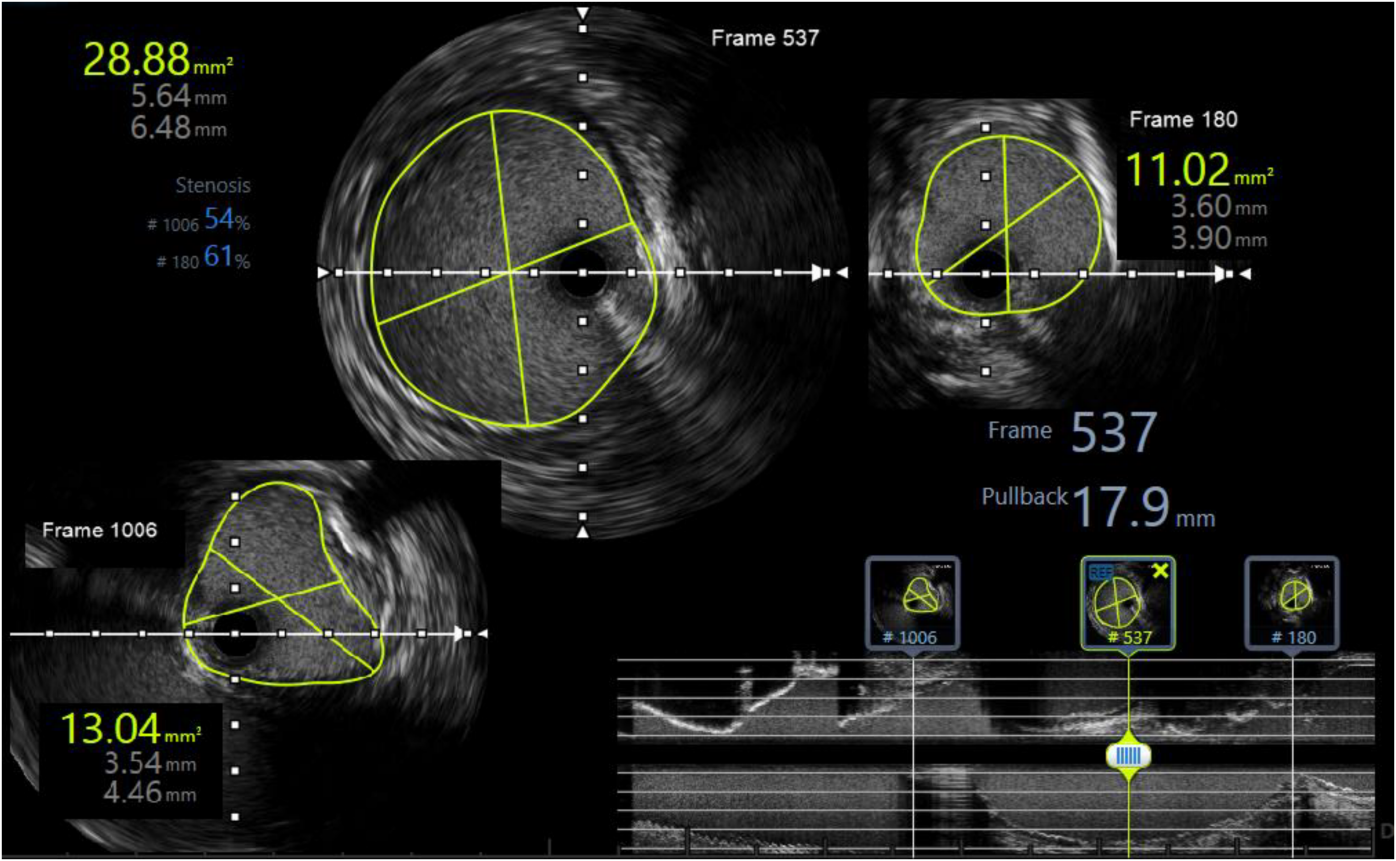
IVUS at ostium ILA (bottom left) shows a cross-sectional area of 13.4 mm^2^ (46% area compared with the middle segment with a cross-sectional area of 28.8 mm^2^) and an anastomosis location stenosis of 61% caused by atherosclerosis.

On the first postoperative day, the patient developed oliguria, and a Doppler ultrasonography (US) of the renal vessels was performed, following the hospital protocol of kidney transplantation. Ultrasound showed significant stenosis at the grafted renal artery–internal iliac artery with a peak systolic velocity of 198 cm/s and an intrarenal impedance index in the upper third, the middle, and the bottom at 0.51, 0.57, and 0.63, respectively. After the interdisciplinary consultation, digital subtracted angiography (DSA) was indicated to confirm the diagnosis. We approached from the right brachial artery with a Slender 7F size sheath. Pigtail imaging revealed moderate stenosis of the ostium internal iliac artery and anastomosis site. There was a suspected dissection of the graft renal artery just distal to the anastomotic site as well. IVUS was used (Figures 2, 3), with a JR 4.0 7F Launcher guide (Medtronic), a 0.014-inch run-through wire (Terumo), and OptiCross 18 3.5F 135 cm IVUS (Boston Scientific). Ultimately, we identified a non-significant lesion at the ostium of the internal iliac artery and a significantly reduced vascular area compared to the midsegment. Additionally, at the midsegment where the anastomosis was, there was atherosclerotic plaque resulting in moderated lumen stenosis and dissection flap immediately distal to the junction with an arc 180° and 4.5 mm long, and 61% stenosis of the artery was not an absolute indication for intervention. But with the dissection and acute kidney injury that came along, the indication became clear.

**Figure 4:**
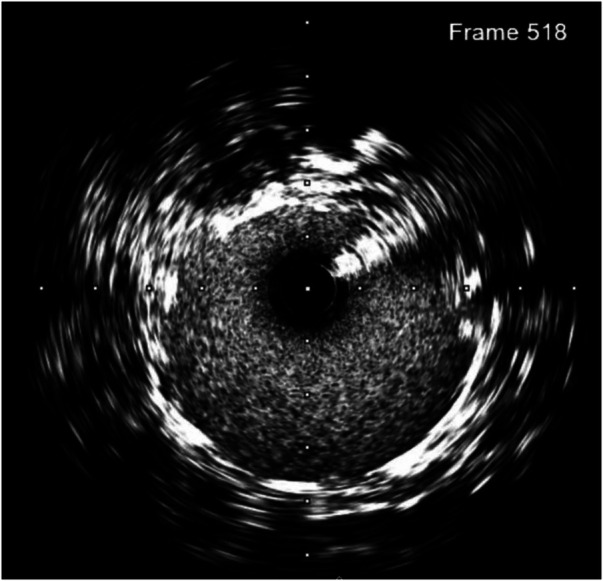
Post-intervention IVUS showed improved minimal lumen area, with no stent edge dissection.

To prevent the progression of dissection and improve perfusion of the transplanted kidney, we decided to deploy a 5.0 × 22 mm Resolute Onyx stent crossing over the narrowing anastomosis and dissected site, followed by balloon angioplasty with 5.0 × 12 mm Pantera LEO balloon at 20 atm pressure. A DES was chosen to address two problems at a time, which were atherosclerosis lesions with 61% plaque burden and the dissection complication. The 5.0 × 22 mm Resolute Onyx stent was the most appropriate choice regarding the length and diameter based on the lab availability at that time. The 20 atm pressure was chosen because, with the above IVUS images, 18 atm pressure would not fit the vessel wall. The dissection was covered by one stent, and the minimal lumen area was improved to 23 mm^2^ on the IVUS image. The fluoroscopy time was 14 min, and the used contrast was 120 ml of Visipaque 320. Post-intervention IVUS is presented in Figure 4.

After the intervention, Doppler US revealed that the blood flow of the renal artery was adequate without an increase in systolic blood velocity, resulting in sufficient blood flow supplied for the allograft. Kidney function improved after 3 weeks, and the patient was discharged after 4 weeks from the transplantation. Normal urine output returned after 3 weeks, and serum creatinine level decreased to 1.19 mg/dl after 2 months. The cause of slow recovery was attributed to acute tubular necrosis.

Postprocedural medications included dual antiplatelet with aspirin 81 mg and plavix 75 mg for 1 month followed by maintenance of a single antiplatelet with aspirin 81 mg. An immunosuppression regimen with tacrolimus, mycophenolate mofetil, and methylprednisolone was prescribed. He was closely monitored at an outpatient clinic and continued to be in stable condition after 4 years.

## 3 Discussion

As far as is currently understood, complications following renal transplantation are uncommon, with dissection of the graft renal artery being particularly rare. In the event that it does occur, balloon dilatation or surgery is the treatment of preference, which is usually performed after months of observation (5–8). We documented a case in which the graft renal artery was dissected at an extremely early stage; stenting was effectively executed, yielding favorable results during the follow-up periods for both inpatients and outpatients. We also performed IVUS to determine the exact mechanism of the incident, which was the dissection.

As shown in Table 1, the incidence of vascular complications following transplantation is exceedingly low, with renal artery stenosis being the most prevalent. Dissection was observed to occur at a low rate. However, certain advancements have been made in the management approach: balloon dilatation and surgery have been supplemented with stenting, and IVUS has been implemented both prior to and following stenting. Short-term and long-term follow-up appeared to indicate that the intervention produced outstanding results in every case, from the earliest case (3 days in our case) to months after transplantation.

**Table 1:** Studies evaluating complications and management for dissection after renal artery transplantations.

Study	Indication for intervention	Time to intervention	Type of intervention	Outcomes and follow-up
1. Raynaud et al. (5): case series	- 41 patients: stenosis of the surgical anastomosis of the main renal artery distal to the anastomosis – 7 patients: stenosis of a renal artery branch	–	Balloon catheter dilatation	- 81% immediate postprocedural successful in angiography - 3 immediate complications: 1 dissection, 1 perforation, 1 peripheral embolus - 7 patients: restenosis before the 5th month following PTA
2. Merkus et al. (9)	2 cases of iliac artery dissection	6 months in case 1	- Surgery: Dacron bypass in case 1 - No reported intervention in case 2	- Case 1: blood pressure and renal function were stable at the 16-year follow-up - Case 2: stable during follow-up
3. Peregrin et al. (10)	Renal artery dissection	3 days	Angioplasty and stenting	Stable during follow-up
4. Delles et al. (11)	Iliac artery dissection	5 days	Stenting	Stable renal function during follow-up
5. Takahashi et al. (12)	3 patients with renal artery dissection	Within a week	Angioplasty and stenting	- 2 cases were stable during follow-up - 1 case experienced sepsis and hemorrhagic arterial infarctions on the first day post-intervention
6. Saito et al. (13)	Renal artery dissection	During the surgery	Angioplasty	Stable during follow-up
7. Humke et at. (14)	3 patients with renal artery dissection	–	Angioplasty and stenting	The interventions were successful Follow-up: 2 cases were stable in urine output and creatinine concentration; 1 case had peripheral arterial thrombosis
8. Dar et al. (15)	5 cases of external iliac artery dissection	During the surgery	PTFE interposition graft	Successful surgery and stable during follow-up
9. Kırnap et al. (16)	2 cases of external iliac artery dissection	During the surgery	Failed percutaneous angioplasty -> reconstructed surgery	Both patients remain stable
10. Karusseit (17)	External Iliac artery dissection	During the surgery	Surgery: reconstruction	Stable 2-year follow-up
11. Lushina et al. (18)	External iliac artery dissection	During the surgery	Open endarterectomy	Stable follow-up to date
12. Vijayvergiya et al. (19)	Left external iliac artery dissection	Postoperative period	Stenting	Stable 2-year follow-up
13. Hori et al. (20)	Renal artery dissection	During the surgery	Surgery: re-anastomosis	Urine output restored and serum creatinine decreased
14. Vijayvergiya et al. (21)	Left external iliac artery dissection	2 months	Balloon dilatation and stenting	- Serum creatinine decreased to 1.2 mg/dl at 2-week follow-up - The patient was asymptomatic at 1-year follow-up
15. Vijayvergiya et al. (7): a case report	Dissection at the anastomotic site of the transplanted renal artery	2 months	- Stenting with IVUS before and after intervention	Improved urine output; better-controlled blood pressure Serum creatinine resolved at 3-month follow-up period
16. Zhang et al. (8): single-center	Renal artery stenosis	3.7 ± 2.7 months	- Balloon catheter dilatation (3) - Stenting (33)	- The overall treatment of renal artery rupture, thrombosis, renal artery kinking, and other complications is poor - In the case of renal artery dissection, normal renal function was restored.
	External iliac artery dissection	<1 day (*n* = 4), 3 months (*n* = 1)	- Artificial revascularization (3) - Bypass (1) - Covered stent (1)	
	Renal artery disruption	14.5 ± 2.5 days	- Nephrectomy (3) - Repatch (1)	
	Renal vein thrombosis	11.0 ± 3.6 days	Nephrectomy	
	Renal artery thrombosis	7 days	Nephrectomy (1), PTA, Thrombolysis (1)	
	Renal artery dissection	3 days	PTA with covered stent	
	Renal artery kinking	2 days	Nephrectomy	
	Pseudoaneurysm of the internal iliac artery	6 months	PTA	
	Pseudoaneurysm of the renal artery	3 months	PTA	
17. Sharma et al. (22)	External iliac artery dissection	During the surgery	Balloon thrombectomy and endarterectomy	Good renal allograft function until 4 years postoperatively
18. Mayhew et al. (23)	Renal artery dissection	During the surgery	Arterial reconstruction	Creatinine continued to decline at follow-up, with excellent kidney function
19. Bayraktar et al. (24)	External iliac artery dissection	During the surgery	Surgery: internal iliac artery transposition	- Doppler ultrasound after surgery: RI value increased in the proximal common femoral artery; compatible with stenosis up to 50% with the monophasic flow - 6-year follow-up serum creatinine was 0.7 g/dl
20. Our study (2024)	Dissection of the graft renal artery distal to the anastomotic site	3 days	Stenting with IVUS before and after intervention	- Urine output gradually returned after 3 weeks, and serum creatinine level was normalized after 2 months - Clinically and laboratory stable at 4-year follow-up

From the perspective of an endovascular interventionist, we identified three problems based on this case.

The first issue to be concerned about was the difference in stenosis severity of the internal iliac artery in the two imaging modalities. While pretransplant MRI only revealed a slight narrowing at the ostium, the DSA image showed a moderate-severe stenosis. The explanation proposed for this situation was the kinking of the artery. There was a change in the axis of the internal iliac artery before and after the surgery. This was confirmed by IVUS imaging, which shows a non-significant lesion at the ostium of the internal iliac artery and a significantly reduced vascular area compared with the midsegment. In addition, when the right kidney is transplanted into the left iliac fossa, it often has a longer artery than the grafted vein, leading to torsion and angulation of the artery (25).

The second problem is the narrowing at the site of anastomosis of the grafted renal artery. In literature, the majority of TRAS is located distal to the anastomosis site (26–28). Transplant artery stenosis or post-anastomotic stenosis is due to disease of the transplant renal artery itself. This site is consistent with endothelial proliferation. On the other hand, recipient artery stenosis or pre-anastomotic stenosis was caused by trauma due to vessel loops or clamps during transplantation or, more often, due to neglected atheroma in the recipient artery. Lesions in the internal iliac artery were most commonly observed. Stenosis at the suture line or anastomotic stenosis was caused by technical suture errors: i.e., excessive bites or tightening of continuous suture lines. The difference in texture and caliber between the internal iliac and renal artery increases the incidence of such technical mishaps. In this case, we suggest that the narrowing of the anastomosis was caused by differences in vessel wall stiffness because, during the suturing process, we discovered atherosclerosis and calcification of the iliac artery at the anastomosis site, while the wall of the graft renal artery was normal.

The third problem is the dissection distal to the anastomosis site. On the IVUS image, we saw the dissection of the endothelium with 180° arc and 4.5 mm length. To prevent thrombosis, we placed a stent to cover this lesion. According to the literature, thrombosis of the graft artery is very rare with an incidence of 0.5%–3.5%, but its consequences are very severe with most cases being loss of the transplanted kidney (3, 4). It is often due to technical difficulties encountered during organ retrieval and implantation, as well as the creation of an intimal flap during donor nephrectomy or perfusion. It is also seen when there is size discrepancy and misalignment, torsion, or kinking of the anastomosis. Proposed risk factors include the presence of atherosclerosis, polycystic kidney disease, and vascular collagen disorder (1, 20, 29, 30). Allograft function after TRAD can potentially be saved with appropriate treatment, including surgical revascularization or stent implantation (3, 4, 29). Even though we could not conclude the cause of the renal artery dissection in this case, there were multiple attributed risk factors, for instance, atherosclerosis and calcified lesions at the anastomosis site, difficulty of suture, and kinking of the anastomosis. In terms of treatment strategy, DSA and endovascular intervention are appropriate options according to current recommendations with a high successful rate (1) and the availability and experiences of endovascular intervention at our hospital. In addition to determining the vascular size and lesion length for stent selection, IVUS also helped us to characterize the nature of the ostium stenosis lesion of the internal iliac artery, which was due to kinking rather than atherosclerosis in this specific case (31).

## 4 Conclusion

This case signified the importance of recognizing the complication phenomena after transplantation, and stenting intervention can be a useful treatment strategy in very early dissection cases. The treatment decision of renal artery dissection after transplantation should be based on the timing of diagnosis, conditions of the transplant kidney and patient, extent of dissection, availability of vascular stents and angiographic resources, and, importantly, surgeons’ expertise. Our patient received endovascular stenting with the help of IVUS image, which was minimally invasive, safe, and effective. In view of the current limited evidence, a multidisciplinary approach should be adopted in the management of iliac artery dissection involving urologists, nephrologists, and endovascular interventionists.

## Data Availability

The raw data supporting the conclusions of this article will be made available by the authors, without undue reservation.
